# Editorial: Metal-based nanomaterials for tumor diagnosis and therapy

**DOI:** 10.3389/fchem.2025.1738897

**Published:** 2025-11-17

**Authors:** Siwen Liu, Jianhua Liu

**Affiliations:** Department of Radiology, Second Affiliated Hospital of Jilin University, Changchun, China

**Keywords:** nanomaterial, metal-based, diagnosis, therapy, tumor

## Introduction

With the rapid development of nanomaterials, metal-based nanomaterials have achieved remarkable progress in recent years and demonstrated great application potential in many fields. A characteristic example of diverse properties is with transition metal nanoparticles: gold nanoparticles, due to their excellent biocompatibility and unique optical properties, are widely used in bioimaging and tumor-targeted therapy, while palladium nanoparticles exhibit outstanding performance in organic synthesis reactions (Sakthi et al., 2022; Yang et al., 2022).

In terms of tumor diagnosis, metal-based nanomaterials possess the ability to enhance imaging contrast. For example, superparamagnetic iron oxide nanoparticles (SPIONS) can significantly improve the resolution of MRI for tumors, facilitating the early detection of tiny lesions (Shaghaghi et al., 2025). Gold nanoparticles, on the other hand, are able to realize the precise localization of tumor cells through surface-enhanced Raman scattering (SERS) technology (Li et al., 2022; Wen et al., 2022). Additionally, certain metal-based nanomaterials can achieve targeted molecular diagnosis; their surfaces can be modified with targeting molecules such as tumor-specific antibodies and nucleic acid probes, which can actively recognize and bind to biomarkers on the surface of tumor cells (Khosroshahi et al., 2022). This targeting ability may reduce interference with normal cells and greatly lower the misdiagnosis rate, making it particularly suitable for the screening of early asymptomatic tumors. In tumor therapy, metal-based nanomaterials play roles in delivering highly targeted drugs and mediating physical therapy. Among them, some metal-based nanomaterials, such as mesoporous silica-coated gold nanoparticles, can serve as drug carriers (Li et al., 2021; Quiñones et al., 2023). They have the ability to encapsulate chemotherapeutic drugs, gene drugs, etc., inside or adsorb them on the surface. Through passive targeting or active targeting, drugs are accurately delivered to tumor sites, reducing the distribution of drugs in normal tissues and lowering toxic side effects.

## Highlights of contributions relating to the Research Topic

We have launched a Research Topic entitled “Metal-based Nanomaterials for Tumor Diagnosis and Therapy,” which aims to promote research on metal-based nanomaterials in the field of tumor diagnosis and therapy. Herein, we hope to receive insights from professionals in the relevant field, focusing on the challenges and current prospects of metal-based nanomaterials. This will facilitate their further development and early application in the diagnosis and treatment of tumor patients. Therefore, we have received and published four peer-reviewed articles (original research and review papers) under this Research Topic.

The first article by Yang et al.systematically reviews the latest application progress of magnetic nanomaterials (MNMs) in the fields of medical imaging and therapy. It focuses on the key technical challenges existing in the application of these materials and the emerging development opportunities for the synergy of imaging and therapeutic functions. By comprehensively integrating the practical achievements of MNMs in multimodal imaging (such as MRI and CT) and diverse therapeutic methods (such as magnetic hyperthermia and targeted drug delivery), this article strives to provide valuable research directions and technical ideas for the optimized design of MNMs in precision medicine scenarios and their in-depth application in personalized treatment regimens.

The second paper by Zhang et al. reports the development of a nanoprobe, WL-12@Fe_3_O_4_, for detecting PD-L1 expression in malignant pleural mesothelioma. This was achieved by conjugating PD-L1-binding peptide (WL-12) with SPIONs. The study evaluated the nanoprobe’s stability, biotoxicity, targeting ability, and *in vivo* MRI imaging performance. Results showed that the WL-12@Fe_3_O_4_ probe exhibited a uniform spherical shape, good dispersibility, and no cytotoxicity or organ damage, demonstrating its broad application prospects.

The next article in this Research Topic by Tian et al.focuses on the application of nanomaterials in cancer immunotherapy, aiming to address issues such as limited efficacy and high systemic toxicity in traditional cancer immunotherapies, including chimeric antigen receptor T (CAR-T) therapy and immune checkpoint blockade therapy. The article first introduces the mechanisms, characteristics, and limitations of traditional immunotherapies. It then elaborates on the properties and advantages of nanomaterials like liposomes, lipid nanoparticles, polymeric nanoparticles, and self-assembled scaffolds, such as improving drug stability, enabling precise targeted delivery and controlled release, and reducing toxicity. It also details the specific applications of these nanomaterials in immunotherapy, for instance, delivering antigens and immune checkpoint inhibitors to enhance immune responses. Finally, it analyzes the prospects and challenges of the clinical translation of nanomaterials, providing references for optimizing cancer immunotherapy strategies and promoting the clinical application of nanomaterials.

We also include another meaningful research article by Ma et al. This study constructs a core-shell structured nanoplatform, IrO_x_@MPN. It uses multi-cage IrO_x_ as the core, with the outer layer coated by a metal-phenolic network (MPN) self-assembled from Fe^3+^ and tannic acid (TA). This nanoplatform is specifically designed for tumor treatment scenarios that adapt to the acidic tumor microenvironment (TME) and excessive intracellular glutathione (GSH). Among its functions, the multi-cage structure of IrO_x_ can synergistically exert photothermal therapy (PTT) effects with chemodynamic therapy (CDT), significantly enhancing tumor suppression efficacy. Meanwhile, IrO_x_@MPN possesses excellent multimodal imaging performance, including CT and T_1_/T_2_-weighted MRI. This performance supports the early diagnosis of tumors and real-time monitoring during treatment, providing a typical reference example for the development of novel multifunctional platforms for dual-response tumor therapy.

## Summary

With the continuous breakthroughs of metal-based nanomaterials in interdisciplinary fields such as materials science and biomedicine, their applications in the field of tumor diagnosis and therapy have become increasingly widespread, bringing brand-new solutions to tumor patients. Endowed with unique physicochemical properties, including controllable size and morphology, excellent biocompatibility, and versatile functional adaptability, metal-based nanomaterials have been expanding the application boundaries in tumor diagnosis and therapy. They have evolved from being mere single-function tools like imaging contrast agents or drug carriers into multifunctional platforms that integrate precise diagnosis, targeted therapy, and therapeutic efficacy monitoring. We hope this Research Topic will serve as a strong driving force for advancing the study of metal-based nanomaterials, inspire deeper reflections on tumor diagnosis and therapy, and accelerate the translation of nanomaterials from basic research to clinical practice at an earlier date.
